# Endoscopically assisted procedure for removal of a foreign body from the maxillary sinus and contemporary endodontic surgical treatment of the tooth

**DOI:** 10.1186/1746-160X-2-37

**Published:** 2006-11-08

**Authors:** Fabio Costa, Massimo Robiony, Corrado Toro, Salvatore Sembronio, Massimo Politi

**Affiliations:** 1 Department of Maxillo-Facial Surgery, Faculty of Medicine, University of Udine, Udine, Italy

## Abstract

There have been reports on the migration of teeth or implants into the maxillary sinus. We know of only one report on the migration of a gutta-percha point that had been used to fill a root canal into the ethmoid sinus. We report such a case treated with an endoscopically assisted procedure for removal of the foreign body and contemporary endodontic surgical treatment of the tooth.

## Findings

A 33-year-old woman was seen for a chronic pain in the region of tooth 26 in June 2005. She had treatment to the root canal of her left upper first molar in 2002. The patient's history indicated also previous symptoms in the last two years of maxillary sinusitis including tenderness in the left infraorbital region and nasal stuffiness.

**Figure 1:**
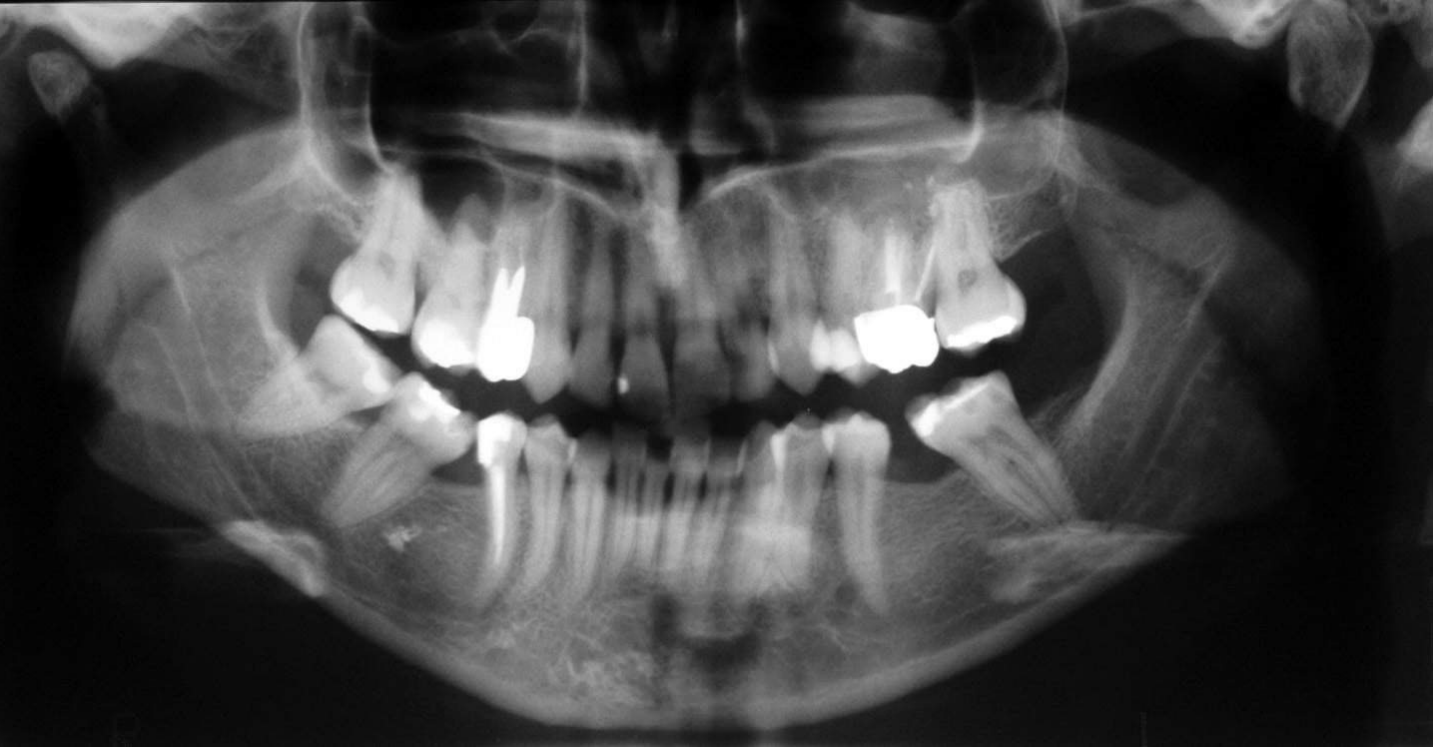
Orthopantomography showing radiopacity of the left maxillary sinus.

**Figure 2:**
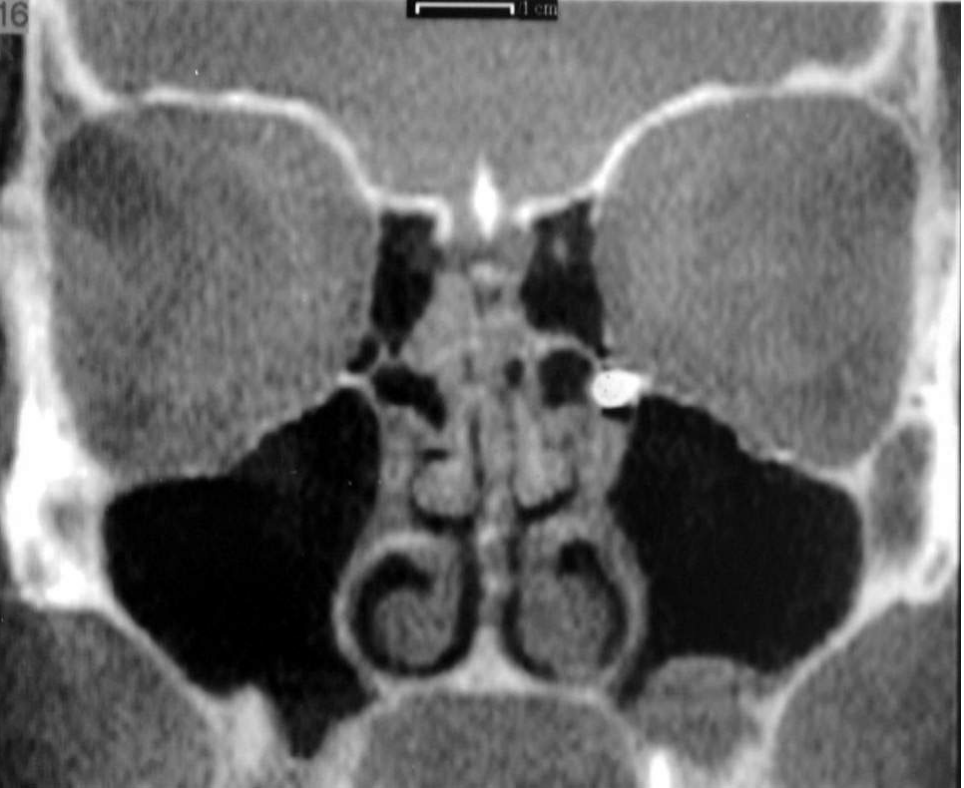
Computed tomography scan (coronal plane) showing the foreign body located in the supero medial aspect of the maxillary sinus and partial mucosal thickening of the sinus upon the roots of the upper first molar.

**Figure 3:**
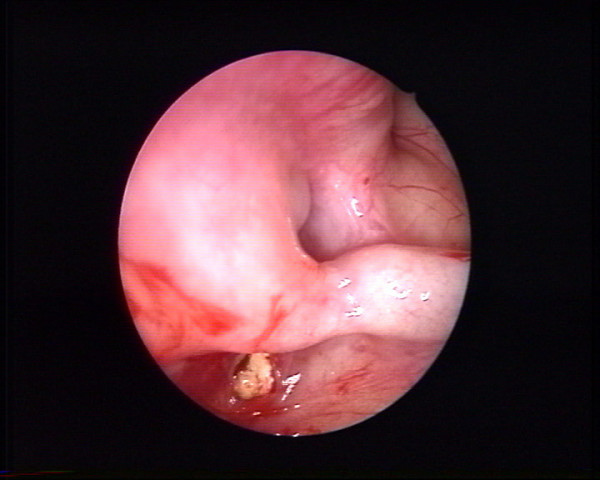
Intraoperative endoscopic view of the foreign body in the supero medial aspect of the maxillary sinus.

**Figure 4:**
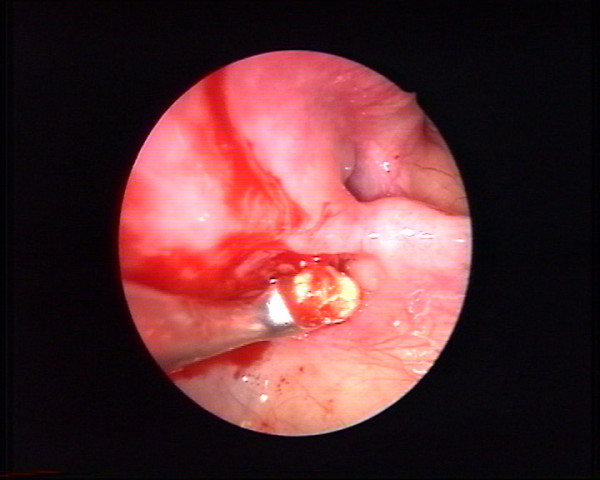
Intraoperative endoscopic view of the foreign body removal.

**Figure 5:**
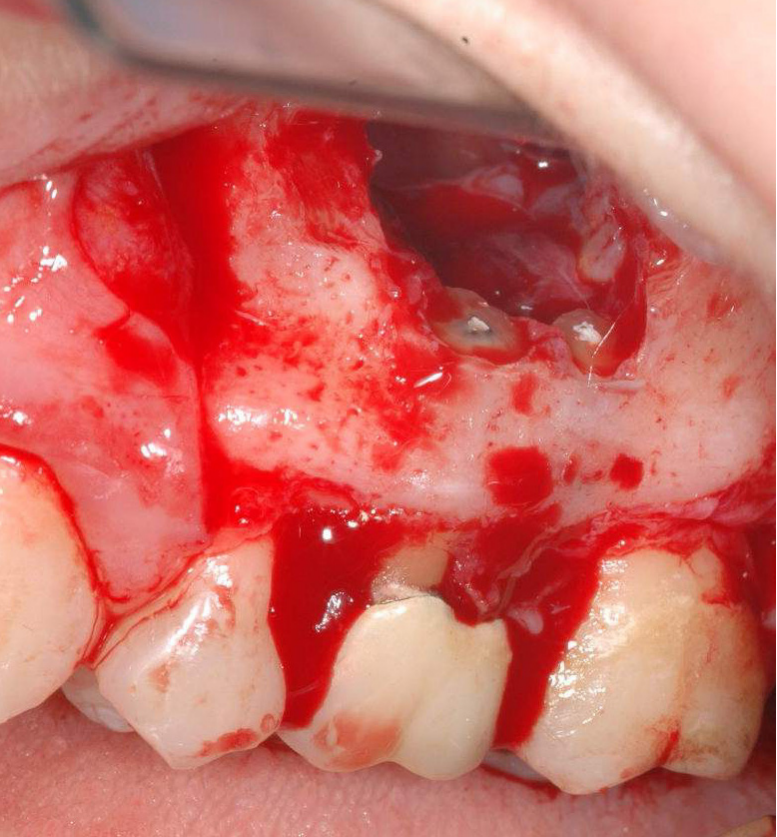
Intraoperative view of contemporary endodontic surgical treatment of the upper first left molar roots.

Clinical examination identified pain in the region of the left upper first molar and an orthopantomography showed a radiopacity of the left maxillary sinus (Fig. 1). Computed tomography showed the presence of a foreign body located in the supero medial aspect of the maxillary sinus, near the natural maxillary ostium and it looked like a residual endodontic cement. A partial mucosal thickening of the sinus upon the roots of the upper first molar was also present (Fig. 2). Videorinoscopy showed hypertrophy of the inferior turbinates bilaterally. The surgical plan was to remove the foreign body with contemporary treatment of the odontogenic source. In July 2005 with the patient under general anesthesia, standard surgical technique was used to create a small osteotomy in the lateral antral wall upon the roots of the upper first left molar. The antrum was examined through the endoscope and the foreign body was easily identified and gently removed (Fig. 3, 4). A contemporary endodontic surgical treatment of the upper first left molar roots and an endoscopic reduction of inferior turbinates were performed (Fig. 5). Direct examination of the foreign body confirmed that it was a residual endodontic cement. Her postoperative course was satisfactory with no evidence of sinus infection.

## Discussion

Removal of foreign bodies through an endonasal endoscopic approach is the treatment of choice [1]. Endoscopically assisted Caldwell-Luc procedure for removal of a surgical bur from the maxillary sinus was also described [2]. There has been no previous report about endoscopic removal of a filling agent migrated from the root canal into the maxillary sinus. Migration through the maxillary sinus of a gutta percha point into the ethmoid sinus was described [3]. In our case, as in the case previously described, it is most likely that the endodontic cement went from the roots of the upper left first molar to the natural ostium by the action of the cilia that continue to clear mucus toward the natural ostium.

It is possible that the foreign body dislocated near the maxillary natural ostium created an antral inflammation of the overlying mucosa and a disturbance in the clearence of the maxillary sinus. This fact with the concomitant hypertrophy of the inferior turbinates may explain the patient's previous symptoms of maxillary sinusitis including tenderness in the left infraorbital region and nasal stuffiness.

In this case a small bone window in the lateral wall of the maxillary sinus was performed in order to obtain a contemporary endodontic surgical treatment of the upper first left molar roots. This report shows how contemporary removal of a foreign body from the maxillary sinus and treatment of the odontogenic source may be obtained through a minimally invasive endoscopically assisted access to the maxillary sinus.

## Competing interests

The author(s) declare that they have no competing interests.

## Authors' contributions

MP and CT designed the study. SS contributed to writing the paper. FC and MR performed surgery and wrote the main part of the paper All authors gave useful comment on the text of the manuscript. Written consent was obtained from the patient or their relative for publication of study.
